# The Impact of Glucose-Lowering Drugs on Sarcopenia in Type 2 Diabetes: Current Evidence and Underlying Mechanisms

**DOI:** 10.3390/cells10081958

**Published:** 2021-08-01

**Authors:** Elena Massimino, Anna Izzo, Gabriele Riccardi, Giuseppe Della Pepa

**Affiliations:** Department of Clinical Medicine and Surgery, Federico II University, Via Sergio Pansini 5, 80131 Naples, Italy; elenamassimino@libero.it (E.M.); ariannaizzo.1991@gmail.com (A.I.); riccardi@unina.it (G.R.)

**Keywords:** glucose-lowering drugs, type 2 diabetes mellitus, sarcopenia, skeletal muscle index, skeletal muscle mass

## Abstract

The age-related decrease in skeletal muscle mass together with the loss of muscle power and function is defined sarcopenia. Mounting evidence suggests that the prevalence of sarcopenia is higher in patients with type 2 diabetes mellitus (T2DM), and different mechanisms may be responsible for this association such as impaired insulin sensitivity, chronic hyperglycemia, advanced glycosylation end products, subclinical inflammation, microvascular and macrovascular complications. Glucose-lowering drugs prescribed for patients with T2DM might impact on these mechanisms leading to harmful or beneficial effect on skeletal muscle. Importantly, beyond their glucose-lowering effects, glucose-lowering drugs may affect per se the equilibrium between protein anabolism and catabolism through several mechanisms involved in skeletal muscle physiology, contributing to sarcopenia. The aim of this narrative review is to provide an update on the effects of glucose-lowering drugs on sarcopenia in individuals with T2DM, focusing on the parameters used to define sarcopenia: muscle strength (evaluated by handgrip strength), muscle quantity/quality (evaluated by appendicular lean mass or skeletal muscle mass and their indexes), and physical performance (evaluated by gait speed or short physical performance battery). Furthermore, we also describe the plausible mechanisms by which glucose-lowering drugs may impact on sarcopenia.

## 1. Introduction

The improvement of healthcare systems and the advancements in the prevention and treatment of major non communicable illnesses, together to the economic and social development, have substantially increased the longevity of the population worldwide.

In 2020, there were 727 million persons aged 65 years or over in the global population. This number is projected to double to 1.5 billion in 2050. Globally, the share of the population aged 65 years or over is expected to increase from 9.3% in 2020 to 16% in 2050 [1].

Although population aging may represent a human success story, it is widely known that aging is associated with a physiological impairment in all areas of the body. Many changes and dysfunctions also occur in skeletal muscle. In fact, a progressive and generalized loss of muscle mass can be observed after the age of 40 years; the rate of deterioration has been estimated to be 8% every 10 years up to 70 years, and 15–25% every 10 years after this age [2]. Similarly, a 10–15% loss of leg strength every 10 years has been reported to occur up to 70 years, increasing to 25–40% every 10 years after this age [3].

These age-related changes in skeletal muscle make up an independent condition termed sarcopenia, now recognized by the International Classification of Disease, Tenth Revision, Clinical Modification (ICD-10-CM), code (i.e., M 62.84) [4]. Sarcopenia is defined as a syndrome in which muscle mass and muscle function (muscle strength and/or physical performance) progressively decrease with age [5,6]. Sarcopenia diagnosis is established by the presence of low muscle quantity or quality. When low muscle quantity/quality, low muscle strength, and low physical performance are all detected, sarcopenia is considered severe. 

The age-related changes involved in sarcopenia not only affect the upper and lower limbs, but rather involve a decline in mass and strength of the respiratory muscles configuring the recent term of “respiratory sarcopenia” [7]. Respiratory sarcopenia is characterized as whole-body sarcopenia with low respiratory muscle mass followed by low respiratory muscle strength and/or deteriorated respiratory function [8]. Various factors may lead to respiratory sarcopenia such as aging, inactivity, undernutrition, diseases, inflammation, and cachexia [8]. The diaphragm is the primary muscle of the respiratory pump, and reduced diaphragm muscle mass impairs inspiratory strength and cough function [8]. The reduction in respiratory muscle function in the elderly makes this population more vulnerable to disease and disability [6,9], and contributes to respiratory complications, a common cause of morbidity and mortality in the elderly [10,11]. 

A wide variety of tests and tools are now available for the characterization of sarcopenia; however, the main diagnostic tools are: (1) clinical questionnaire to confirm clinical suspicions as initial screening, followed by (2) the instrumental measuring of muscle strength and quantity, and finally (3) the detection of muscle performance.

The prevalence of sarcopenia worldwide ranges from 10% to 40%, based on the different characteristics of the population and the different criteria to assess it [12]. 

Different risk factors are involved in the age-related changes characterizing sarcopenia. These include genetic factors, gender, ethnical background, socioeconomic status, as well as modifiable factors such as low physical activity, poor diet [13,14,15], and sleep disturbance [16,17], while the effects of alcohol consumption and cigarette smoking are not clear [18,19,20]. Comorbidities and pharmacological intervention might further contribute to sarcopenia in the elderly [14,15].

The mechanisms underlying the age-related changes involved in sarcopenia are not completely understood and are related to several and interacting processes: decline in the synthesis of muscle proteins [21], post-prandial resistance to synthesize protein in response to various nutritional factors [22], alteration in hormone balance (testosterone, dehydroepiandrosterone, vitamin D, growth hormone, insulin-like growth factor 1 and cortisol) [23], intracellular lipid accumulation in the muscle [24], dysregulation of proteasomal degradation pathways [25], increase in oxidative stress and mitochondrial dysfunction [26], imbalance in proinflammatory cytokines [27], reduction in the number of satellite cells in the muscle [28], low motor unit remodeling [29]. 

Type 2 diabetes mellitus (T2DM) is one of the most common metabolic disease and represents an important health condition for the aging population, affecting one quarter of people over the age of 65 years; this proportion is projected to further increase in the next decades [30]. Individuals with T2DM are at a higher risk of developing sarcopenia [31,32,33].

However, it is possible that by improving the metabolic derangement in these patients by an appropriate treatment, the risk of sarcopenia may be reduced. 

A life-course approach of behavior modification represents a key approach for the prevention and treatment of sarcopenia [15]. It is important to underline that, although the loss of muscle mass appears to be an inevitable part of the aging process, there is variation in the rates of decline across the population [34], indicating that modifiable behavioral factors could influence the development of sarcopenia. For instance, systematic reviews have shown that achieving the recommended levels of physical activity, in particular resistance-based training, is an effective protective strategy against sarcopenia and has a positive impact on muscle mass/strength and physical capacities in elderly [35,36,37,38,39,40]. Resistance-based training is similarly effective for improving muscle strength, size, and quality also in elderly with T2DM [41]. The role of a nutritional intervention for the prevention and treatment of sarcopenia is much less clear, although some evidence shows the benefit of healthier dietary patterns such as adequate intake of protein, vitamin D, antioxidants, and long-chain polyunsaturated fatty acids [42,43,44,45].

Beyond lifestyle changes, it should be considered that several glucose-lowering drugs are available for the treatment of T2DM, and they exert hypoglycemic activity throughout various mechanisms that may impact differently on the pathophysiological derangements leading to sarcopenia. In this respect, the scenery of pharmacological management of patients with T2DM has grown widely complex in the last years.

Guidelines suggest the need to account for heterogeneous characteristics of patients, putting the patients at the center of care, individualizing treatment targets and goals [46,47,48,49].

Currently, the most prescribed glucose-lowering drugs are metformin, thiazolidinediones (TZDs), sulfonylureas, dipeptidyl peptidase-4 inhibitors (DPP-4i), glucagon-like peptide-1 receptor agonists (GLP-1 RAs), sodium-glucose transport protein 2 inhibitors (SGLT2i), and insulin [50].

Against this background, the aim of this narrative review is to provide an update on the effects of glucose-lowering drugs on sarcopenia in individuals with T2DM, focusing on the parameters used to define sarcopenia: muscle strength (evaluated by handgrip strength), muscle quantity/quality (evaluated by appendicular lean mass or skeletal muscle mass or their indexes), and physical performance (evaluated by gait speed or short physical performance battery). Furthermore, we also describe the plausible mechanisms by which glucose-lowering drugs may impact on sarcopenia.

## 2. Materials and Methods

### 2.1. Search Procedures

Literature probing for this narrative review [51] was conducted by searching PubMed database for articles published during the last 20 years (2001–2021), considering that the majority of glucose-lowering drugs has been approved in the last twenty years; however, we also considered, if available, the most relevant studies before this period. The search dated from October 2020 to May 2021.

The medical subject heading (MeSH) terms “sarcopenia” OR “skeletal muscle mass” OR “muscle strength” OR “handgrip strength” OR “appendicular skeletal muscle mass” OR “muscle mass” OR “gait speed” AND “glucose-lowering drugs” OR “antidiabetic drugs” OR “metformin” OR “biguanides” OR “thiazolidinediones” OR “glitazones” OR “sulfonylureas” OR “DPP-4 inhibitors” OR “gliptins” OR “incretins” OR “GLP-1 receptor agonists” OR “SGLT2 inhibitors” OR “gliflozins” OR “insulin therapy” AND “diabetes” were utilized.

The search was limited to humans, clinical trials, and the English language.

An initial exploratory electronic database search was conducted by the two reviewers (E.M. and A.I.) to define the final search terms. Then, both reviewers independently conducted the main research. The herewith identified studies were screened for eligibility using titles and abstracts. The remaining full texts were assessed to ascertain whether they were fulfilling the inclusion and not fulfilling the exclusion criteria.

For the aim of our narrative review—the impact of glucose-lowering drugs on sarcopenia in T2DM—we focused particularly on clinical trials performed in individuals with T2DM, excluding retrospective and cross-sectional studies, meta-analyses, and systematic reviews on epidemiological studies.

Studies that did not meet the selection criteria, duplicate publications, non-original articles, consensus papers, letters to the editor, editorials, and studies in languages other than English were excluded. 

Initially, 978 articles of potential intertest were found, and after screening of title and abstracts, and the assessment of the remaining full texts fulfilling the inclusion criteria, 36 papers were used for the aim of this narrative review.

### 2.2. Sarcopenia Evaluation

Different outcomes related to sarcopenia have been evaluated in the various trials. In particular, the most important parameters are muscle strength, muscle quantity/quality, and physical performance. The skeletal muscle strength is usually evaluated by a calibrated handheld dynamometer under well-defined test conditions with interpretive data from appropriate reference populations [5]; the chair stand test is a simple mode of evaluating strength and endurance when a disability comprising the handgrip evaluation is present [5]. The muscle quantity/mass has been reported as total body Skeletal Muscle Mass (SMM), as Appendicular Skeletal Muscle Mass (ASM) or as muscle cross-sectional area of specific muscle groups or body locations. Because SMM/ASM strictly correlate with body size, they can be adjusted for height or for body mass index (BMI)—namely using height squared, weight or body mass index—resulting in skeletal muscle mass indexes (SMI). The major SMI used are ASM/height^2^ and SMM/height^2^. 

Muscle quantity/mass has been evaluated by different techniques such as: Magnetic Resonance Imaging (MRI), Computed Tomography (CT), Dual-energy X-ray absorptiometry (DXA) [52,53]. Finally, bioelectrical impedance analysis (BIA) has been explored for estimation of total SMM or ASM indirectly by estimating muscle mass based on whole-body electrical conductivity [54].

Muscle performance has been measured by different methods such as gait speed, the Short Physical Performance Battery (SPPB), the Timed-Up and Go test (TUG), and the 400 m walk test. The gait speed test that is usually performed is called the 4 m usual walking speed test and requires the participant to move 4/6 m at a usual walking speed [55]. The SPPB is a composite test that includes assessment of gait speed, a balance test, and a chair stand [56]. For the TUG test, individuals are asked to rise from a standard chair, walk to a marker 3 m away, turn around, walk back, and sit down again [57]. The 400 m walk test assesses walking ability and endurance. For this test, participants are asked to complete 20 laps of 20 m [5]. 

## 3. Effects of Glucose-Lowering Drugs on the Parameters of Sarcopenia

### 3.1. Metformin

Evidence from epidemiological studies indicates that men with T2DM using insulin sensitizers, such as metformin and TZDs, lost significantly less SMM (−1.1 vs. −2.9%) or ASM (−1.8 vs. 4.4%) than those treated without insulin sensitizers [58]. Similarly, in a cohort of 2864 women with T2DM, patients taking insulin sensitizers had less decline in usual walk speed than those not taking insulin sensitizers (−1.07 vs. −0.10 m/s), without significant differences in grip strength [59]. However, patients with T2DM and sarcopenia were significantly less likely to receive biguanides [60,61,62,63]. 

Although evidence from epidemiological studies show that metformin might have beneficial effect on parameters of sarcopenia, data from clinical trial are less consistent. Aghili et al. reported that in patients newly diagnosed with T2DM and placed on metformin (1000 mg/twice daily) for 24 weeks, men showed a greater significant increase in SMI (+2.6%) than women; interestingly, in women, a reduction in the ASM (−4%) was observed [64] (Table 1). In a prospective open-label observational study, overweight patients with T2DM and chronic obstructive pulmonary disease, treated with metformin (850 mg/twice daily) for 24 weeks, showed a significant reduction in handgrip strength (−3.2%) [65].

In contrast, in patients with T2DM treated with the DPP-4i sitagliptin randomized to receive the SGLT2i ipragliflozin (50 mg/daily) or metformin (1000 mg/twice daily) for 24 weeks, there were no significant differences between the ipragliflozin and metformin groups regarding changes in abdominal muscle area (−2.9% vs. −1.9%, respectively) and handgrip strength (+0.9% vs. +8.8%, respectively). However, analysis performed in the elderly population revealed that handgrip strength was significantly higher than that in the non-elderly following metformin treatment (+8.8% vs. +0.3%, respectively) [66].

In support of a possible beneficial influence of metformin on sarcopenia, there are also data from a clinical trial performed in elderly individuals without T2DM showing a significant improvement in mean walking time (by 0.39 s) with this drug; this value corresponds to an improvement of the mean gait speed of 0.13 m/s [88]. Importantly, it should be considered that all the reported trials suffer of important methodological limitations of, such as a small sample size, a relatively short trial duration and an open label design.

### 3.2. Thiazolidinediones

As reported above, epidemiological studies show that TZDs in patient with T2DM were associated with a significantly lower reduction in muscle mass and usual walk speed [58,59]. In contrast, data from clinical trials are scarce and conflicting. Furthermore, the few trials available were performed in individuals without T2DM.

Yokota et al. investigated the effects of the TZD pioglitazone (15 mg/daily) in individuals at risk for T2DM. After 16 weeks of treatment there was no significant change in the cross-sectional area of the calf muscle (57.9 vs. 58.2 cm^2^). Notably, pioglitazone reduced the intra-myocellular lipid content, improved skeletal muscle fatty acid metabolism, and decreased the muscle phosphocreatine loss during exercise, indicating improved skeletal muscle high-energy phosphate metabolism [89]. Bastien et al. evaluated the effect of rosiglitazone vs. placebo on aerobic exercise capacity and body composition/distribution in men with T2DM and stable coronary artery disease. After one year, patients randomized to rosiglitazone showed no significant changes in lean mass and total mid-thigh SMM (−1 vs. −5 cm^2^) [90].

In contrast, a trial performed in individuals without T2DM has shown that men who were given pioglitazone lost more thigh muscle volume, compared to men who were not given pioglitazone; however, muscle resistance training was effective in counteracting muscle loss [91]. Furthermore, pioglitazone was able to potentiate the effect of resistance training on muscle strength in women but not in men [92]. 

Further well-designed trials are needed to elucidate the effects of TZDs on sarcopenia in patients with T2DM.

### 3.3. Sulfonylureas

Data from the Food and Drug Administration-Adverse Effects Reporting System database show that during an 8-month observational period, muscle atrophy was found in 0.27% of the glibenclamide/glyburide reports in humans and in 0.02% of the other non-sulfonylureas drugs [93]. 

In a cross-sectional study, Rizzo et al. observed that sarcopenic parameters (SMM, SMI, muscle strength, and gait speed) were significantly worse in patients with T2MD taking sulfonylureas compared with DPP-4i [94], while in a clinical trial no changes in SMM were reported in patients with T2DM assigned to sulfonylureas [67]. 

### 3.4. Dipeptidyl Peptidase-4 Inhibitors

Epidemiological evidence shows that elderly diabetic patients treated with DPP-4i have better parameters of sarcopenia compared with those treated with other glucose-lowering drugs [94].

Again, Ishii et al. reported that SMM significantly increased in overweight/obese patients with T2DM assigned to the DPP-4i sitagliptin (+2.6%) compared with sulfonylurea (−0.7%) after 24 weeks of treatment [67]. In a randomized, multicenter, controlled study, patients with T2DM were assigned to receive the DPP-4i sitagliptin (50 mg/daily) or the SGLT2i ipragliflozin. After 12 weeks, a significant decrease in SMI (−0.10 kg/m^2^) was observed only in the ipragliflozin group, while no changes in the sitagliptin group were reported [72]. Similarly, in overweight/obese patients with T2DM, no changes in SMM and SMI were reported following 24 weeks of the DPP-4i teneligliptin (20 mg/daily) compared with the GLP-1 RAs dulaglutide; during the latter drug, a reduction in both the evaluated markers of sarcopenia was observed [68] (Table 1). Although scarce, these few data suggest that DPP-4i, beyond their proven efficacy and safety in terms of glucose control in elderly patients with T2DM [46,47,48,49], might be a good choice with respect to the risk of sarcopenia due to their neutral impact.

### 3.5. Glucagon-Like Peptide-1 Receptor Agonists

Six months of the GLP-1 RAs dulaglutide treatment (0.75 mg/weekly) compared with the DPP-4i teneligliptin (20 mg/daily) significantly reduced SMI (−1.2%) in patients with T2DM treated with insulin and on hemodialysis. Furthermore, in the dulaglutide group SMM was significantly decreased (−3.8%) [68] (Table 1). However, it is important to underline that in this study the participants were more likely to develop sarcopenia, considering the age—higher than in other studies—and the presence of complications related to advanced renal failure in all patients [68].

Conversely, Perna et al. assessed the effect of the GLP-1 RAs liraglutide (3 mg/daily) on SMM changes in overweight and obese elderly patients with T2DM. After 24 weeks, a worsening in the parameters of sarcopenia was not observed in any patients under treatment; importantly, five patients even showed an improvement in SMI [69]. Another trial shows that liraglutide over 24 weeks induced a non-significant reduction in SMM (−0.6%) [70]. Similarly, Hong et al. did not report significant changes in SMM (+4.3%) after 12 weeks of treatment with the GLP-1 RAs exenatide (20 μg/twice daily) [71] (Table 1).

### 3.6. Sodium-Glucose Transport Protein 2 Inhibitors

In recent years, many trials focusing on the effects of SGLT2i on the parameters of sarcopenia have been performed. A study conducted in Japanese patients with T2DM showed that the treatment with SGLT2i (ipragliflozin 50 mg, luseogliflozin 2.5 mg, or dapagliflozin 5/10 mg daily) after 10 weeks increased grip strength [73]. In patients with T2DM and non-alcoholic fatty liver disease (NAFLD), dapagliflozin (5 mg/daily) significantly decrease SMM (−2.1%) compared with the standard treatment after 24 weeks [74]. In contrast, Sugiyama et al. showed that SMI did not significantly decrease in overweight patients with T2DM after treatment with dapagliflozin (5 mg/daily) compared with non SGLT2i (−0.8 vs. −8.0%); furthermore, both treatments produced a non-significant reduction in SMM (−0.7%) [75]. Similarly, Yamakage et al. showed no differences in SMM in obese patients with T2DM after treatment with dapagliflozin (5 mg/daily) compared with non-SGLT2i (+0.4 vs. −0.7%) [76]. On the other hand, in patients with T2DM and non-alcoholic steatohepatitis, dapagliflozin (5 mg/daily) promoted a significant increase in ASM (+2.2%), without changes in SMM (+0.4%) and SMI (+1.2%) after 24 weeks [77].

Luseogliflozin (5 mg/day) induced a significant reduction in SMI at and after 36 weeks (−1.9%) in obese patients with T2DM [78]. Similarly, overweight patients with T2DM treated with luseogliflozin (5 mg/day) showed a significant reduction in SMM (−2.2%) and SMI (−2.9%) after 12 weeks [79].

In a prospective single-arm study, patients with T2DM and NAFLD were treated with canagliflozin (100 mg/day). After 56 weeks, a not significant slight decrease in SMM (−0.4%) was observed [80].

Overweight patients with T2DM receiving insulin treatment were randomly assigned to add-on ipragliflozin (50 g/day) or no additional treatment. After 24 weeks, the change in SMM was somewhat larger in ipragliflozin than in the control group, but the difference was not significant (+0.1 vs. −1.2%) [81]. In contrast, another trial with ipragliflozin (50 mg/once daily) reported a significant reduction in SMI (−2.7%) by week 8 and this reduction remained at the end of the study [82]. 

In a multicenter prospective Japanese study, patients received ipragliflozin (50 mg/daily) as monotherapy or combined with other glucose-lowering drugs for up to 104 weeks shown a significant reduction in SMM (−0.9 kg) [83].

Similarly, a significant reduction in SMM (-0.6 kg) following ipragliflozin treatment (50 mg/daily) for 12 weeks was observed in a small trial [84]. In contrast, Miyake et al. find a not significant reduction in SMM (−2.2%) after 24 weeks of treatment with ipragliflozin (50 mg/daily) in patients with T2DM and NAFLD [85].

Matsuba et al. investigated the impact of tofogliflozin (20 mg/daily) in patients with T2DM showing a significant decrease in SMM (−1.4 kg) after 12 weeks of treatment [86].

Summarizing, clinical trials consistently show that reduction in SMI and SMM can occur during treatment with SGLT2i [72,74,78,79,82,83,84,86], and muscle mass loss may represent a major concern with the use of SGLT2i, especially in elderly and lean patients, (Table 1). However, in one trial an improvement in grip strength [73] and ASM [77] was reported, (Table 1).

Finally, in six trials no significant changes on SMI and SMM were reported [75,76,77,80,81,85] (Table 1).

Further studies evaluating the effects of SGLT2i on sarcopenic parameters should be considered, particularly in normal weight elderly patients, in good metabolic control, also considering a larger sample size and various ethnic backgrounds.

### 3.7. Insulin

Epidemiological evidence suggests that patients with T2DM and sarcopenia are more frequent insulin users [95]. A retrospective observational study reported that insulin treatment prevented the decline of SMI in the lower extremities [96].

In 118 participants with T2DM of the population-based KORA-Age study, during a follow-up of three years, the SMI decline was significantly more marked in patients treated with oral glucose-lowering drugs compared to insulin therapy, suggesting a positive effect of insulin treatment on muscle mass. In particular, women on insulin therapy displayed a significantly greater increase in time needed to complete the TUG than women treated with oral glucose-lowering drugs (2.6 vs. 0.7 s, respectively), despite more favorable—albeit not statistically significant—changes in SMI over time compared to those treated with oral glucose-lowering drugs (+0.1 vs. −0.3 kg/m^2^, respectively). In men, the change in SMI differed significantly between participants treated with insulin compared with those treated with oral glucose-lowering drugs (+0.2 vs. −0.3 kg/m^2^, respectively), whereas changes in TUG did not differ in men with or without insulin therapy. Changes in gait speed were not significantly differed in women or men with and without insulin therapy [87] (Table 1).

## 4. Type 2 Diabetes Mellitus and Sarcopenia

Type 2 diabetes mellitus (T2DM) represents an important health burden in the elderly population, affecting approximately 25% of people over the age of 65 years; this proportion is expected to further increase in the next decades [30]. Increasing attention has been paid to sarcopenia in individuals with T2DM for its heavy impact on the quality of life of elderly patients with T2DM [97], in whom frailty and sarcopenia—in addition to the traditional microvascular and macrovascular complications of diabetes—are emerging as a third category of complications leading to disability [98]. A higher prevalence of sarcopenia has been consistently reported in T2DM (ranging from 5 to 50%) than in individuals without T2DM [31,32,33]. 

The risk factors related to sarcopenia in elderly people with T2DM are age, diabetes duration, gender, presence of comorbidity, microvascular complications [32,99], reduced hip circumference, lower BMI, low physical activities [100], and poor nutritional status [101,102].

Different mechanisms may explain the higher prevalence of sarcopenia in individuals with T2DM. The anabolic effects of insulin on the skeletal muscle are well known; their pathways involve phosphoinositide3-kinase, phosphoinositol triphosphate, protein kinase B/AKT, TBC1 domain family member 4 and 1. In addition to the synthesis of protein, these pathways induce the translocation of glucose transporter type 4 (GLUT4) containing vesicles to the plasma membrane [103], and the dis-inhibition of glycogen synthesis by phosphorylation of glycogen synthase kinase 3, leading to an improvement in muscle metabolism. AKT also induces the activation of mTOR, which promotes protein synthesis, and inhibits fork head box protein O activity, thus reducing the expression of E3 ubiquitin ligases that mediate the atrophy of muscle cells [104]. All these pathways are progressively impaired by the insulin resistance associated with T2DM that may lead to a decrease in protein synthesis and an increased protein degradation, thus promoting loss in muscle mass and strength [105]. Again, insulin resistance decreases muscle mass and muscle contractility due to the increase in inflammatory cytokines, myostatin expression, together with the reduction in blood flow and skeletal muscle glucose uptake [106]. Interestingly, patients with a longer duration of T2DM are more likely to decrease muscle mass and strength predominantly in the lower extremities rather than in the upper extremities [107,108], probably due to a reduction in numbers of the predominantly oxidative fiber type [109].

Chronic hyperglycemia in T2DM promotes per se an increase in advanced glycosylation end products (AGEs) in skeletal muscle that might affect muscle mass, grip strength, leg extension power, and walking speed [110]. AGEs may increase oxidative stress, inflammatory cytokines, and promote the formation of cross-links and breakdowns of muscular proteins in elderly individuals [111,112]. Importantly, when blood glucose levels are high, a large amount of glucose is channeled into the polyol pathway, resulting in enhanced formation of sorbitol which is converted by sorbitol dehydrogenase to fructose. The metabolites derived from the polyol pathway reduce cellular antioxidant defense, promote the formation of AGE precursor (methylglyoxal) via auto-oxidation, and activate the protein kinase C pathway via de novo synthesis of diacylglycerol. These phenomena collectively promote oxidative stress and inflammation [113].

In T2DM an increase in low-grade inflammation and some inflammatory cytokines has been reported [114]. Available evidence shows that inflammatory cytokines, such as tumor necrosis factor-α, interleukin-6, and C reactive protein, may negatively affect muscle mass, strength and function by promoting muscle atrophy via blunting muscle anabolism and energy homeostasis, and promoting muscle protein degradation via the NF-kB pathways [27,30,98]. Oxidative stress is strictly related to low grade inflammation; increasing evidence suggests that oxidative stress plays a pivotal role in the pathophysiology of insulin resistance and T2DM [115,116], as well as in age-related muscle changes and sarcopenia by affecting skeletal muscle size, fiber activation, excitation-contraction coupling, and cross-bridge cycling [117,118,119].

Finally, the presence of microvascular—retinopathy, nephropathy, and neuropathy—and macrovascular—cardiovascular diseases—diabetic complications may further explain the higher prevalence of sarcopenia in T2DM. In particular, retinopathy and the associated visual impairment may impact on balance, involved in both locomotion and physical performance, causing mobility limitations that might affect physical activity [120], muscle strength and quality [121]. Nephropathy may contribute to sarcopenia, in particular promoting muscle loss, through metabolic acidosis, mitochondrial dysfunction, increased inflammation, protein loss, reduced vitamin D synthesis, defects in insulin/insulin-like growth factor 1 intracellular signaling, inflammation and catabolic responses [122]. Peripheral diabetic neuropathy is characterized by the accelerated loss of motor units resulting in muscle weakness, atrophy, and intramuscular fatty infiltration that favor loss of muscle strength, power, and endurance [123,124]. Macrovascular complications, such as peripheral vascular disease, may favor muscle ischemia, as well as lower muscle strength, mass, and performance [125].

## 5. Glucose-Lowering Drugs and Mechanisms of Their Potential Impact on Sarcopenia

Several glucose-lowering drugs for the treatment of T2DM have become available in the last years. All glucose-lowering drugs for T2DM can beneficially impact on some of the mechanisms possibly involved in sarcopenia; in fact, they improve blood glucose control and insulin sensitivity, inhibit AGEs formation, and improve inflammation and the oxidative stress. Moreover, glucose-lowering drugs such as TZDs, GLP-1 RAs and SGLT2i have been shown also to reduce the incidence and progression of microvascular and macrovascular complications [46,47,48,49]. On the other hand, some of them—metformin, GLP-1 RAs, and SGLT2i—have a potential detrimental effect on the risk of sarcopenia since they help to decrease body weight by reducing energy intake or increasing glucose excretion by glycosuria, negatively impacting on muscle mass. For contrasting reasons, sulfonylureas and insulin, which favor weight gain, can also increase the risk of sarcopenia, since this can aggravate insulin resistance and related mechanisms involved in the pathogenesis of sarcopenia. Furthermore, beyond their glucose-lowering effects, glucose-lowering drugs may affect per se the equilibrium between protein anabolism and catabolism through several mechanisms involved in skeletal muscle physiology, thus contributing to sarcopenia; however, data on this aspect are unclear or controversial, and human studies in most cases are lacking. 

### 5.1. Metformin

Metformin is the most widely prescribed drug for the treatment of T2DM and it promotes a glucose-lowering effect by the activation of the AMP-activated protein kinase (AMPK) pathway that leads to an inhibition of hepatic gluconeogenesis. Furthermore, metformin acts on skeletal muscle by increasing insulin-stimulated glucose uptake, and on gastrointestinal tract promoting gut microbiome changes, intestinal glucose uptake, and secretion of gastrointestinal hormones [126]

**Figure 1:**
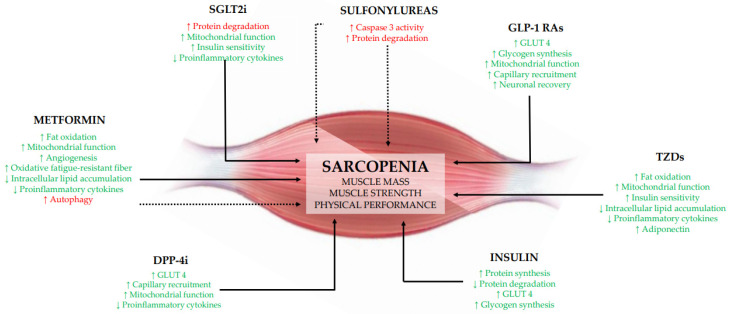
Plausible mechanisms by which glucose-lowering drugs might impact on sarcopenia acting on sarcopenia. DPP-4i, dipeptidyl peptidase-4 inhibitors; GLP-1 Ras, glucagon-like peptide-1 receptor agonists; GLUT4, glucose transporter type 4; SGLT2i, sodium-glucose transport protein 2 inhibitors; TZDs, thiazolidinediones. ↑, increase; ↓, decrease. Green color and continue lines indicate a beneficial effect on sarcopenia; red color and dotted lines indicate a detrimental effect on sarcopenia.

The possible beneficial effects of metformin on parameters of sarcopenia observed in the few clinical trials performed so far could be related to different mechanisms (Figure 1). Metformin could act on muscle by activating AMPK and stimulating gene expression of peroxisome proliferator-activated receptor-γ coactivator 1-α to upregulate the transcription of genes involved in fatty acid oxidation, thus reducing muscle lipid accumulation; furthermore, the activation of the AMPK pathway could stimulate angiogenesis, increase mitochondrial biogenesis, and switch muscle fiber types from glycolytic to more oxidative fatigue-resistant fibers [127,128,129]. Furthermore, the inhibition of NF-κB signaling by AMPK activation could suppress the inflammatory response, which contributes to depress muscle protein breakdown [130]. On the other hand, it has been also observed that in the human muscle metformin reduces the expression of mTORC1-related genes in older individuals with impaired glucose tolerance [131], leading to a possible decrease in muscle protein synthesis or increased autophagy [132].

### 5.2. Thiazolidinediones

TZDs improve insulin sensitivity through activation of the peroxisome proliferator-activated receptor-γ which facilitates differentiation of mesenchymal stem cells into adipocytes, promotes lipogenesis in peripheral adipocytes, decreases hepatic and peripheral triglycerides, decreases activity of visceral adipocytes, and increases adiponectin production. These primary effects of TZDs markedly ameliorate insulin resistance, decreasing insulin requirement and positively impact on the features of the metabolic syndrome [133].

TZDs might increase plasma levels of adiponectin and expression of its receptors, activates AMPK and acetyl-CoA carboxylase in muscle, and increases expression of genes involved in mitochondrial function and fat oxidation, promoting an improvement in mitochondrial respiratory capacity in skeletal muscle [134]. The expression of these genes may lead to a decrease in toxic intracellular lipid metabolites, thus improving the insulin signaling in muscle and enhancing insulin sensitivity [135] (Figure 1).

### 5.3. Sulfonylureas

Sulfonylureas increase the release of insulin through the stimulation of pancreatic β-cells. By binding to a subunit of potassium ATP-dependent channels, which consists of a sulfonylurea receptor-1 and an inward-rectifier potassium ion channel, this class of glucose-lowering drugs inhibit the cellular release of potassium, leading to cell depolarization. As a result, an inflow of calcium into the cell occurs which causes an increase in insulin exocytosis [136].

Looking at possible mechanisms, we can only speculate using data from in vitro studies. Sulfonylurea induced atrophy in rat skeletal muscles by reducing the protein content in the flexor digitorum brevis [93]; the activation of the atrophic signaling was induced through caspase-3-dependent or independent pathways [137] (Figure 1).

### 5.4. Dipeptidyl Peptidase-4 Inhibitors

DPP-4i are glucose-lowering drugs that rapidly and specifically inhibit DPP-4 activity. DDP-4 is the main enzyme involved in the biochemical pathways affecting both the degradation and inactivation of the gastrointestinal hormones GLP-1 and gastric inhibitory polypeptide (GIP). By preventing this, DPP-4i enhance active GLP-1 and GIP levels by 2 to 3-fold following a meal, promoting the secretion of insulin and the inhibition of glucagon release [138].

The mechanism by which DPP-4i may increase muscle mass is unclear and could be related to their ability of enhancing GLP-1 action or to the inhibition of DPP-4 activity per se or both. In animal models, DPP-4i promoted GLUT4 expression in the soleus and gastrocnemius muscles [139] and improved skeletal muscle glucose uptake by enhancing capillary recruitment and interstitial insulin concentrations [140]. Furthermore, an improvement in mitochondrial biogenesis and exercise capacity in skeletal muscle has also been reported [141]. Finally, there is evidence that DPP-4i reduce plasma levels of the inflammatory parameters in humans [142] (Figure 1).

### 5.5. Glucagon-Like Peptide-1 Receptor Agonists

GLP-1 RAs bind with high specificity to the GLP-1 receptor and stimulate glucose-dependent insulin release from the pancreatic β-cells. GLP-1 improves the glycemic control by enhancing both synthesis and secretion of insulin via the glucose-dependent pancreatic insulin pathways; it also induces, via a paracrine route, the inhibition of glucagon secretion from pancreatic α-cells. Other effects of GLP-1 are the slowing down of the rate of endogenous glucose production, the retardation of gastric emptying, thus preventing large post-meal glycemic increments, the promotion of satiety, and the reduction in the caloric intake and body weight [143].

The potential mechanisms regarding the effect of GLP-1 RAs on skeletal muscle remain a matter of debate. GLP-1 RAs could increase the expression of GLUT4 gene, glucose uptake, and glycogen synthesis in skeletal muscle cells [144]. Again, GLP-1 RAs promote oxygen consumption and favor insulin sensitivity in skeletal muscle [145]. Furthermore, the infusion of GLP-1 RAs increases microvascular recruitment in human skeletal muscle independent of insulin, which may potentiate local insulin action [146]. Interestingly, GLP-1 RAs might facilitate neuronal recovery after the crush nerve, leading to an improvement in muscle health [147] (Figure 1).

### 5.6. Sodium-Glucose Transport Protein 2 Inhibitors

The SGLT2 are carrier proteins expressed in the proximal convoluted tubule of the kidney, where they significantly contribute to the reabsorption of approximately 90% of renal glucose. Thus, SGLT2i exert their glucose-lowering effects by reducing the renal threshold for glucose reabsorption and inducing glucose urinary excretion. However, they increase hepatic glucose production, glucagon secretion, ketogenesis, and lipid oxidation, which may promote reduction in body fat mass, including visceral fat [148]. Muscle mass loss may represent a major concern with the use of SGLT2i, and different mechanisms may explain this effect: the reduction in insulin levels and increase in glucagon levels promoted by SGLT2 lead to the activation of gluconeogenesis, which might promote not only lipolysis in the adipose tissue, but also proteolysis in the skeletal muscle suppling amino acids to the liver and leading to sarcopenia [148]. However, the effect of SGLT2i in the long term may improve insulin sensitivity counteracting muscle catabolism and affecting skeletal muscle function and quality [149]. With respect to the beneficial effects of SGLT2i on maximal handgrip strength, a possible mechanism might be the effects on chronic inflammation and adipokine profile [150], as well as improvement in mitochondrial function [151] (Figure 1).

### 5.7. Insulin

Epidemiological evidence and the clinical trial available suggest the beneficial association between insulin use and parameters of sarcopenia [87,95,96], indicating that a possible prescription of exogenous insulin could improve insulin signaling in the skeletal muscle, promote protein synthesis, and protect against the loss of muscle mass among patients with diabetes, especially those with a long duration of diabetes.

Insulin is an anabolic hormone that increases protein synthesis in the muscle, promotes glucose uptake and glycogen synthesis [152] (Figure 1). Furthermore, physiological hyperinsulinemia stimulates muscle protein synthesis and anabolism in young individuals [153]. This effect is blunted in older people and in those with insulin resistance [153], suggesting that supraphysiological hyperinsulinemia could be necessary for the anabolic effect of insulin in elderly individuals [154].

## 6. Conclusions

Prevention of muscle quantity/quality loss might represent an important aim for elderly patients with T2DM, both in relation to the relatively high frequency of sarcopenia, and in consideration of the impact of this condition on the quality of life. 

Prevention of sarcopenia is a major area of research activity, and observational epidemiological studies have identified important risk factors such as older age, genetic factors, gender, ethnical background, low socioeconomic status, low physical activity, poor diet [13,14,15], sleep disturbance [16,17], comorbidities and pharmacological intervention [14,15]. Further risk factors in elderly people with T2DM were diabetes duration and microvascular complications [32,99].

As lifestyle factors are reversible, while age-related systemic changes are largely unmodifiable, it is important to raise public awareness of their impact on the development of sarcopenia.

Physical activity, in particular resistance-based training, and good nutrition represent the cornerstones of healthy ageing [15]. Although the effects of nutritional interventions on muscular outcomes in elderly people have been less carefully evaluated than those of exercise, more data are accumulating in support of the capacity of appropriate nutrition to positively influence muscle mass and function, both alone and in combination with exercise training [15,155]. Evidence, to date, points towards important roles for an adequate intake of protein, vitamin D, antioxidants, and long-chain polyunsaturated fatty acids [42,43,44,45]. 

Beyond lifestyle changes, in recent years, interest of clinicians, nutritionists and scientists has been particularly focused on the role played by glucose-lowering drugs in the modulation of sarcopenia in people with diabetes. In fact, information on this issue may be relevant in order to better identify patients at higher risk for this condition, and to implement appropriate changes in the choice of the glucose-lowering drug, together with lifestyle modifications, in order to reduce the risk of sarcopenia in those who are particularly prone to develop it and, in particular, elderly patients. 

Different glucose-lowering drugs are available for the treatment of T2DM, and the cornerstone of diabetes pharmacological approach is represented by the ability to account for the heterogeneous characteristics of the patients, individualizing treatment targets and goals. Among these goals, increasing attention has been paid to the need to reduce the risk of sarcopenia in elderly patients in whom this condition is emerging as a third category of diabetes complications that can lead to a clinical and significant disability.

The evidence presented in this narrative review shows that glucose-lowering drugs could play a role in sarcopenia in T2DM patients with some differences between them in relation to their mechanisms of action. 

However, beyond a drug-/class-specific effect we cannot exclude a possible effect of glucose-lowering drugs on markers of sarcopenia mediated also by a better glycemic control. In this regard, in the few controlled studies available, the improvement in glycemic control at the end of treatment was similar among the various treatment groups, suggesting that the observed effect on the parameters of sarcopenia could be related to the drug/class specific effect [66,67,68,72,74,75]. On the other hand, in one trial, the improvement of glycemic control in the SGLT2i vs. control was not followed by an improvement in SMM [81]. Again, in two studies without a control group a correlation analysis between the improvement in glycemic control and the observed effect on the parameters of sarcopenia didn’t show statistical significance [78,84]. Therefore, although it seems reasonable to assume that the impact of glucose-lowering drugs on markers of sarcopenia may be partly mediated by an improvement in blood glucose control, the available evidence still supports a specific and direct role of some of them independently of their glucose-lowering activity.

At this point, it is important to underline the limitations of studies discussed in this review, such as the single arm design of many trials, the small sample size, the absence of information regarding dietary habits and physical activity of the participants that could impact on sarcopenia, and the large inhomogeneity of the study populations in terms of age and BMI. Furthermore, parameters of sarcopenia were secondary outcomes in some studies. Importantly, many different methods were used to diagnose sarcopenia that are hardly comparable with each other. The muscle quantity/mass has been reported in the various studies as total SMM, as ASM or as muscle cross-sectional area of specific muscle groups or body locations. In order to overcome this limitation, as seen in Table 1, we focused on SMI—calculated as ASM/height^2^ or SMM/height^2^—or SMM evaluated by dual-energy X-ray absorptiometry or bioelectrical impedance analysis in all trials. For the skeletal muscle strength, we have reported data coming from standardized evaluation performed by a calibrated handheld dynamometer.

Considering all the methodological problems linked to the identification of reliable parameters for the diagnosis of sarcopenia, and the inaccuracies in the design of the available trials, there are only few conclusions that are reliable enough to support clinical recommendations. In particular, it seems well established that insulin is able to reduce the risk of sarcopenia, while DPP-4i might have a neutral impact on it. Data on other glucose-lowering drugs are controversial and inconclusive. However, emerging new data indicate that treatment with SGLT2i may contribute to a reduction in SMI and SMM. These untoward effects of SGLT2i at the muscle level contrast with the multiple beneficial effects of this drug on other relevant and frequent diabetes complications at the level of the kidney and the heart [46,47,48,49].

Nevertheless, on the basis of the evidence reviewed in this paper, it seems appropriate to consider limiting the use of SGLT2i in elderly patients with sarcopenia or at high risk for this condition, unless other clinical conditions justify the need to use them; in this case, a very accurate monitoring of the parameters that characterize sarcopenia would be necessary in order to be able to implement an appropriate treatment, consisting in physical activity, in particular resistance-based training, and good nutrition, in the presence of any derangement.

Further well-designed trials, with large sample sizes, including representative cohorts of elderly patients and with a sufficiently long duration are needed to better elucidate the effects of glucose-lowering drugs on sarcopenia; obviously, for these studies, it is of paramount importance that they take into account all the clinical/instrumental features and pathophysiological mechanisms relevant for the evaluation of this condition. A further interesting aspect to explore should be the impact of a multifactorial intervention combining physical activity and appropriate dietary choices with the most suitable glucose-lowering drugs on muscle mass and function in elderly patients with T2DM, in the perspective that this approach may have a clinically relevant impact on the prevention of sarcopenia in elderly patients at risk for this condition.

## Figures and Tables

**Table 1:** Clinical trials on the effects of glucose-lowering drugs on the parameters of sarcopenia in patients with type 2 diabetes mellitus.

Reference	Study Population	Study Design	Intervention	Duration	Parameters of Sarcopenia	Observed Effects
**Metformin**						
[64]	Participants: 21 M, 30 W Age: 53 ± 10 years BMI: 28.9 ± 3.8 kg/m^2^ HbA1c: 8.2 ± 2.0%	prospective, uncontrolled	Metformin (2000 mg/daily)	6 months	SMI secondary outcome	↓ 0.31 kg/m^2^ *
[65]	Participants: 11 M, 6 W Age: 61 ± 16 years BMI: 29.1 ± 14.9 kg/m^2^ HbA1c: 6.0 ± 1.1%	prospective, uncontrolled	Metformin (1700 mg/daily)	6 months	Handgrip strength secondary outcome	↓ 1.0 kg
[66]	Participants: 14 M, 15 W Age: 69 ± 3 years BMI: 26.9 ± 3.9 kg/m^2^ HbA1c: 8.0 ± 0.8%	prospective, multicenter, randomized, controlled	Metformin (500 mg/daily) vs. Ipragliflozin (50 mg/daily)	6 months	Handgrip strength secondary outcome	↑ 0.25 kg ^§^
**Dipeptidyl Peptidase-4 Inhibitors**						
[67]	Participants: 11 M, 9 W Age: 52 ± 8 years BMI: 29.7 ± 3.2 kg/m^2^ HbA1c: 8.2 ± 1.3%	prospective, controlled	Sitagliptin (50 mg/daily) vs. Glimepiride (1 mg/daily)	6 months	SMM ^†^ secondary outcome	↑ 1.24 kg
**Glucagon-Like Peptide-1 Receptor Agonists**						
[68]	Participants: 16 M, 5 W Age: 68 ± 11 years BMI: 23.1 ± 5.5 kg/m^2^ HbA1c: n.a.	prospective, controlled	Dulaglutide (0.75 mg/weekly) vs. Teneligliptin (20 mg/daily)	6 months	SMI primary outcome	↓ 0.10 kg/m^2^
[69]	Participants: 6 M, 3 W Age: 68 ± 4 years BMI: 32.3 ± 4.9 kg/m^2^ HbA1c: 7.9 ± 1.8%	prospective, uncontrolled	Liraglutide (3 mg/daily)	6 months	SMI primary outcome	↑ 0.03 kg/m^2^
[70]	Participants: 16 M, 12 W Age: 58 ± 10 years BMI: 34.1 ± 5.4 kg/m^2^ HbA1c: 8.3 ± 1.5%	prospective, uncontrolled	Liraglutide (3 mg/daily)	6 months	SMM primary outcome	=
[71]	Participants: 32 M/W Age: 49 ± 11 years BMI: 32.9 ± 4.7 kg/m^2^ HbA1c: 8.7 ± 1.7%	prospective, uncontrolled	Exenatide (20 μg/twice daily)	3 months	SMM secondary outcome	=
**Sodium-Glucose Transport Protein 2 Inhibitors**						
[72]	Participants: 81 M, 38 W Age: 54 ± 11 years BMI: 28.6 ± 5.7 kg/m^2^ HbA1c: 7.5 ± 1.4%	multicenter, controlled, randomized,	Ipragliflozin (50 mg/daily) vs. Sitagliptin (50 mg/daily)	3 months	SMI secondary outcome	↓ 0.10 kg/m^2^
[73]	Participants: 92 M, 20 W Age: 63 ± 10 years BMI: 24.5 ± 4.3 kg/m^2^ HbA1c: 7.0 ± 1.3%	prospective, uncontrolled	Ipragliflozin 50 mg, luseogliflozin 2.5 mg, or dapagliflozin 5/10 mg daily	2.5 months	Handgrip strength primary outcome	↑ ~1 kg
[74]	Participants: 34 M, 23 W Age: 57 ± 12 years BMI: 27.9 ± 4.1 kg/m^2^ HbA1c: 7.8 ± 3.1%	randomized, controlled trial	Dapagliflozin (5 mg/daily) vs. standard treatment	6 months	SMM secondary outcome	↓ 0.9 kg
[75]	Participants: 36 M, 24 W Age: 56 ± 8 years BMI: 27.1 ± 2.9 kg/m^2^ HbA1c: 7.7 ± 0.5%	prospective, controlled	Dapagliflozin (5 mg/daily) vs. oral glucose-lowering drugs	6 months	SMI primary outcome	=
[76]	Participants: 25 M, 29 W Age: 60 ± 13 years BMI: 31.0 ± 6.9 kg/m^2^ HbA1c: 7.5 ± 0.9%	randomized, controlled trial	Dapagliflozin (5 mg/daily) vs. oral glucose-lowering drugs	6 months	SMM secondary	=
[77]	Participants: 9 M, 7 W Age: 53 ± 16 years BMI: 31.0 ± 2.2 kg/m^2^ HbA1c: 7.4 ± 0.5%	prospective, uncontrolled	Dapagliflozin (5 mg/daily)	6 months	SMI secondary outcome	=
[78]	Participants: 27 M, 10 W Age: 54 ± 8 years BMI: 28.0 ± 3.4 kg/m^2^ HbA1c: 7.7 ± 0.7%	prospective, uncontrolled	Luseogliflozin (5 mg/daily)	12 months	SMI primary outcome	↓ 0.15 kg/m^2^
[79]	Participants: 12 M, 5 W Age: 52 ± 12 years BMI: 28.2 ± 2.7 kg/m^2^ HbA1c: 7.5 ± 0.7%	prospective, uncontrolled	Luseogliflozin (5 mg/daily)	3 months	SMI secondary outcome	↓ 0.23 kg/m^2^
[80]	Participants: 11 M, 9 W Age: 51 ± 9 years BMI: 31.5 ± 8.0 kg/m^2^ HbA1c: 8.7 ± 1.4%	prospective, uncontrolled	Canagliflozin (100 mg/daily)	12 months	SMM secondary outcome	=
[81]	Participants: 27 M, 21 W Age: 60 ± 11 years BMI: 27.8 ± 4.2 kg/m^2^ HbA1c: 8.2 ± 0.8%	randomized, controlled trial	Ipragliflozin (50 mg/daily) vs. control	6 months	SMM secondary outcome	=
[82]	Participants: 8 M, 16 W Age: 52 ± 12 years BMI: 28.9 ± 5.4 kg/m^2^ HbA1c: 7.7 ± 0.7%	prospective, uncontrolled	Ipragliflozin (50 mg/daily)	4 months	SMI primary outcome	↓ 0.02 kg/m^2^
[83]	Participants: 234 M, 217 W Age: 55 ± 12 years BMI: 29.4 ± 5.3 kg/m^2^ HbA1c: 8.0 ± 1.4%	multicenter, prospective	Ipragliflozin (50 mg/daily)	24 months	SMM secondary outcome	↓ 0.87 kg
[84]	Participants: 16 M, 4 W Age: 52 ± 10 years BMI: 29.0 ± 5.0 kg/m^2^ HbA1c: 8.5 ± 1.0%	prospective, uncontrolled	Ipragliflozin (50 mg/daily)	3 months	SMM secondary outcome	↓ 0.57 kg
[85]	Participants: 2 M, 10 W Age: 62 ± 15 years BMI: 27.7 ± 5.0 kg/m^2^ HbA1c: 7.6 ± 1.1%	prospective, uncontrolled	Ipragliflozin (50 mg/daily)	6 months	SMM secondary outcome	=
[86]	Participants: 9 M, 5 W Age: 58 ± 10 years BMI: 28.9 ± 4.6 kg/m^2^ HbA1c: 8.2 ± 0.7%	prospective, uncontrolled	Tofogliflozin (20 mg/daily)	3 months	SMM secondary outcome	↓ 1.37 kg
**Insulin**						
[87]	Participants: 60 M, 58 W Age: 76 ± 6 years BMI: 30.5 ± 4.2 kg/m^2^ HbA1c: 6.5 ± 0.6%	prospective, controlled	Insulin vs. oral glucose-lowering drugs	36 months	SMI secondary outcome	↑ 0.15 kg/m^2^

M, men; W, women; BMI, Body mass index; HbA1c, glycated hemoglobin A1c; SMI, skeletal muscle index; SMM, skeletal muscle mass; n.a., not available. =, no significant changes; * in women; ^§^ in the elderly; ^†^ fat and bone-free mass. SMI and SMM were evaluated by dual-energy X-ray absorptiometry or bioelectrical impedance analysis. SMI was calculated as appendicular skeletal muscle mass/height^2^ or skeletal muscle mass/height^2^^.^

## Data Availability

Not applicable.
